# miR-124–3p target genes identify globus pallidus role in suicide ideation recovery in borderline personality disorder

**DOI:** 10.1038/s44184-023-00027-w

**Published:** 2023-06-05

**Authors:** Macarena S. Aloi, Guillermo F. Poblete, John Oldham, Michelle A. Patriquin, David A. Nielsen, Thomas R. Kosten, Ramiro Salas

**Affiliations:** 1 Menninger Department of Psychiatry, Baylor College of Medicine, Houston, TX, USA.; 2 The Menninger Clinic, Baylor College of Medicine, Houston, TX, USA.; 3 Michael E DeBakey VA Medical Center, Houston, TX, USA.; 4 Department of Neuroscience, Baylor College of Medicine, Houston, TX, USA.; 5 Center for Translational Research on Inflammatory Diseases, Michael E DeBakey VA Medical Center, Houston, TX, USA.; 6 These authors contributed equally: Macarena S. Aloi, Guillermo F. Poblete.

## Abstract

Borderline personality disorder (BPD) is characterized by patterns of unstable affect, unstable interpersonal relationships, and chronic suicidal tendencies. Research on the genetics, epigenetics, and brain function of BPD is lacking. MicroRNA-124–3p (miR-124–3p) was recently identified in a Genome-Wide Association Study as likely associated with BPD. Here, we identified the anatomical brain expression of genes likely modulated by miR-124–3p and compared morphometry in those brain regions in BPD inpatients vs. controls matched for psychiatric comorbidities. We isolated lists of targets likely modulated by miR-124–3p from TargetScan (v 8.0) by their preferentially conserved targeting (Aggregate P_CT_ > 0.99, see Supplementary Table 1). We applied Process Genes List (PGL) to identify regions of interest associated with the co-expression of miR-124–3p target genes. We compared the gray matter volume of the top region of interest co-expressing those genes between BPD inpatients (*n* = 111, 46% female) and psychiatric controls (*n* = 111, 54% female) at The Menninger Clinic in Houston, Texas. We then correlated personality measures, suicidal ideation intensity, and recovery from suicidal ideation with volumetrics. Gene targets of miR-124–3p were significantly co-expressed in the left Globus Pallidus (GP), which was smaller in BPD than in psychiatric controls. Smaller GP volume was negatively correlated with agreeableness and with recovery from suicidal ideation post-treatment. In BPD, GP volume may be reduced through miR-124–3p regulation and suppression of its target genes. Importantly, we identified that a reduction of the GP in BPD could serve as a potential biomarker for recovery from suicidal ideation.

## INTRODUCTION

Borderline personality disorder (BPD) is characterized by a pattern of behavioral dysregulation of impulsivity or self-harm, unstable interpersonal relationships, affective instability, distorted self-image, and chronic suicidal tendencies^1^. While the prevalence of BPD in the general population is 1.6%, BPD encompasses a significant portion of the outpatient psychiatric population (10%) and inpatient psychiatric population (20%)^2^. The clinical features of BPD—like depression, anxiety, and suicidal ideation—overlap significantly with other psychiatric disorders^3^. Patients with BPD experience high rates of significant comorbidities with mood, anxiety, substance use, and eating disorders (e.g., up to 96% for lifetime mood disorders; and in cross-sectional studies, 0–35% for generalized anxiety disorder, 2–48% for panic disorder, 3–46% for social phobia, 0–20% for obsessive-compulsive disorder, and 25–56% for PTSD)^4^. In addition, 8–10% of patients die by suicide^5^. Psychiatric disorders originate from the interaction of predisposing genetic, epigenetic, and environmental factors^6^. The etiology of BPD is multifaceted, with significant interactions between genetic, neurological, and psychosocial factors^7^. Most genetic studies have focused on genes within the dopaminergic, serotonergic, and noradrenergic systems^7^. A better understanding of the brain regions and genetic/epigenetic alterations that modulate the risk of BPD will aid in the design of improved and personalized treatments for this condition.

MicroRNAs (miRNAs) are a class of conserved, non-coding ribonucleic acid (RNA) of ~22 nucleotides in length^8^ that regulate gene expression at the post-transcriptional level through translational inhibition or mRNA degradation. In consequence, miRNAs regulate protein expression^9^, potentially affecting brain region function or anatomy through the regulation of specific groups of genes in specific brain regions and cells. A single miRNA can target hundreds of mRNAs and influence the expression of many genes often involved in functional interacting pathways^9^. A variety of miRNAs have been identified to act as regulators of brain development and function, including neural lineage, subtype determination, plasticity, synapse formation, neurogenesis, and the size of substructures in the brain such as the basal ganglia components of the caudate, putamen, and globus pallidus^8^. Prados et al. aimed to determine whether childhood maltreatment—one of the main etiological factors for BPD—was associated with epigenetic changes in subjects with BPD by characterizing the global methylation status of DNA from peripheral blood leukocytes^10^. They found that methylation status near miR-124–3p was lower in persons with BPD relative to patients with major depressive disorder (MDD), suggesting increased expression of this miRNA in BPD^10^.

Given that miR-124–3p regulates the expression of hundreds of genes, we set out to identify brain regions where genes with highly conserved miR-124–3p binding sequences are significantly co-expressed using our Process Genes List (PGL) approach^11–14^. The principal idea behind PGL is that groups of genes that are important to a certain brain-related phenotype must be expressed in the brain region of interest (ROIs) associated with that phenotype^13,15^.

From source lists of genes, PGL identifies the brain regions in reference brains from the Allen Brain Atlas, where those genes are significantly co-expressed. As with most psychiatric conditions, BPD presents with frequent comorbidities; therefore, it is impossible to study biomarker specificity when comparing patients with a psychiatric disorder with healthy controls^16,17^. To circumvent this issue, we utilized a comparison group composed of psychiatric inpatient controls (PC) in the same clinic matched not only for age and sex, but also for comorbid psychiatric disorders^17,18^.

In this study, we aimed to identify the brain regions most likely to be affected by miR-124–3p function, with the hypothesis that those brain regions could be altered in patients with BPD. Using brain morphometry, we identified the left Globus Pallidus (GP) as altered in BPD compared to PC. We then correlated GP volume with the Big Five Inventory (BFI) personality measures of agreeableness and neuroticism, and with recovery from suicidal ideation in BPD compared to our PC cohort.

## METHODS AND MATERIALS

### Healthy controls

Healthy controls (HC, *n* = 111 from a total sample of 141) were recruited from the community. HC participants were required to not have a history or current diagnosis of a mental illness and no contraindications for magnetic resonance imaging (MRI). We used the MINI International Neuropsychiatric Interview for screening^19^. The average age of HC was 26.5 ± 5.75 years and 46% male and 54% female.

### Psychiatric controls, ethics statements, miRNA analysis, and clinical measures

PC (*n* = 111 from a total sample of 407) were recruited from The Menninger Clinic in Houston, Texas, as a part of the McNair Initiative for Neuroscience Discovery—Menninger/Baylor (MIND-MB) research study^16,20^. Inpatient participants were eligible if they did not have MRI contraindications and were considered mentally stable enough to provide informed consent. PC had a variety of psychiatric conditions, including anxiety, mood, personality, and substance use disorders. Over 80% of participants in the PC cohort were diagnosed with comorbid psychiatric conditions. Participants provided signed informed consent. Procedures were approved by the Baylor College of Medicine IRB. Participants stayed at The Menninger Clinic for several weeks (M = 53.5 days, SD = 12.9 days) while receiving medications, psycho-educational groups, 24-h nursing care, individual and group therapy, addictions management if needed, and structured interpersonal and recreational activities. The MIND-MB study collected demographic, clinical, and neuroimaging data, of which relevant data (age, gender, and psychiatric diagnoses from the Structured Clinical Interview for DSM-IV disorders axis I and II^21^) were used in the current study. The BFI measure of personality, consisting of the personality traits neuroticism, extraversion, openness, agreeableness, and conscientiousness, was utilized to conceptualize BPD as maladaptive variants of continuously distributed personality traits in our data^22,23^. The Columbia-Suicide Severity Rating Scale (C-SSRS) was utilized to assess suicidal ideation and behavior in the inpatient setting. For miRNA analysis, see Supplementary Methods.

### Matching control groups to patient groups of interest

Three groups were formed: (1) BPD cohort had a current diagnosis of borderline personality disorder (*n* = 111, taken from the 518 imaged inpatients); (2) PC were without past or current BPD diagnosis, matched to the BPD patients for age, sex, race, and comorbid psychiatric disorders (*n* = 111, taken from the 407 non-BPD inpatients). HC were matched to the BPD group for age, sex, and race (*n* = 111, taken from the 149 possible HC).

An Euclidean distance-matching algorithm was performed to match the HC and PC groups as in ref. ^17^. Demographic characteristics plus psychiatric diagnoses (past and current) were used when matching to PC. To ensure that the groups were not significantly different for any specific feature, a one-way ANOVA for age and Chi-squared tests for all other variables were conducted (*p* < 0.05, no multiple comparisons corrections). This resulted in two groups (BPD and PC) with 111 individuals each (Table 1).

### Neuroimaging: acquisition and analysis

A structural T1-weighted MRI scan was done in a 3T Siemens Trio MR scanner. Scans were acquired at the Core for Advanced MR Imaging at Baylor College of Medicine in Houston, TX. Participants were scanned close to admission to The Menninger Clinic. A ~4.5-min structural MPRAGE sequence was collected (echo time (TE) = 2.66 ms, repetition time (TR) = 1200 ms, flip angle = 12°, 256 × 256 matrix, 160 one-mm axial slices at 1 × 1 × 1 mm voxels).

FreeSurfer version 6.0 was used to perform all preprocessing and automated volumetric segmentation using the T1-weighted structural images. Probabilistic brain mapping was done using FreeSurfer to identify ROIs based on the Aseg atlas^24^ and the Desikan–Killiany Atlas^25^. We divided each patient’s individual ROI volume (in mm^3^) by his/her total Intra Cranial Volume (ICV) to control for ICV.

### Process genes list (PGL) with predicted mRNA targets of miR-124–3p

We isolated a list of target genes likely regulated by miR-124–3p with conserved preferential targeting (>0.99 of Aggregate Pct Score) from Human TargetScan v 8.0^26^. We utilized the list of 93 target genes of miR-124–3p (Supplementary Table 1) and applied PGL (14). PGL was designed to identify brain regions where a group of genes show high levels of co-expression, utilizing the Allen Human Brain Atlas as a reference. The averaged mRNA co-expression of genes of interest is mapped onto brain regions. Using the human Allen Brain Atlas and the PGL GetROIs function^11^ we performed a Wilcoxon test with Bonferroni correction for the number of regions for each brain region identified in at least five of the six brains included in the atlas. This allowed us to find regions in which the difference between the mean mRNA expression levels of miR-124–3p target genes was significantly different from the average of “all other genes.”

### Brain imaging statistics and Big Five Inventory correlations

To avoid multiple comparison problems that are extremely prevalent in brain imaging, we compare BPD to PC on volumetric data of only the top brain region ROI from PGL. Other ROIs were studied on an exploratory basis. We used the BFI inventory to identify possible features that correlate with volumetrics. Specifically, agreeableness and neuroticism have been shown to be the most altered personality characteristics in BPD^22,23^. As intense suicidal ideation is a significant feature of BPD, we additionally interrogated correlations between suicidal ideation recovery as measured by the Columbia-Severity Rating Scale^27^ age, and left GP volume.

### Statistics and correlations

We used a Wilcoxon rank-sum test to compare miR-124–3p levels and left GP volume between PC an BPD groups. Unpaired *t*-tests were used to test differences in lifetime suicidal ideation, ideation at admission, and ideation at discharge (Fig. 4). Hypothesis-driven correlations included (1) left GP volume and agreeableness, or neuroticism, (2) Age and left GP volume, and (3) change in suicidal ideation and left GP volume. Additional exploratory correlations are shown in Pearson’s correlation matrix (Fig. 3A), where other BFI measures of Openness, Consciousness, and Extraversion, were analyzed with left GP volume. Error bars represent the standard error of the mean (SEM). Data were analyzed and graphed in R studio and in GraphPad Prism v 9.2.0.

### Reporting summary

Further information on research design is available in the Nature Portfolio Reporting Summary linked to this article.

## RESULTS

### PGL approach to identify brain regions with the highest co-expression of miR-124–3p target genes identified the Globus Pallidus

**Fig. 1:**
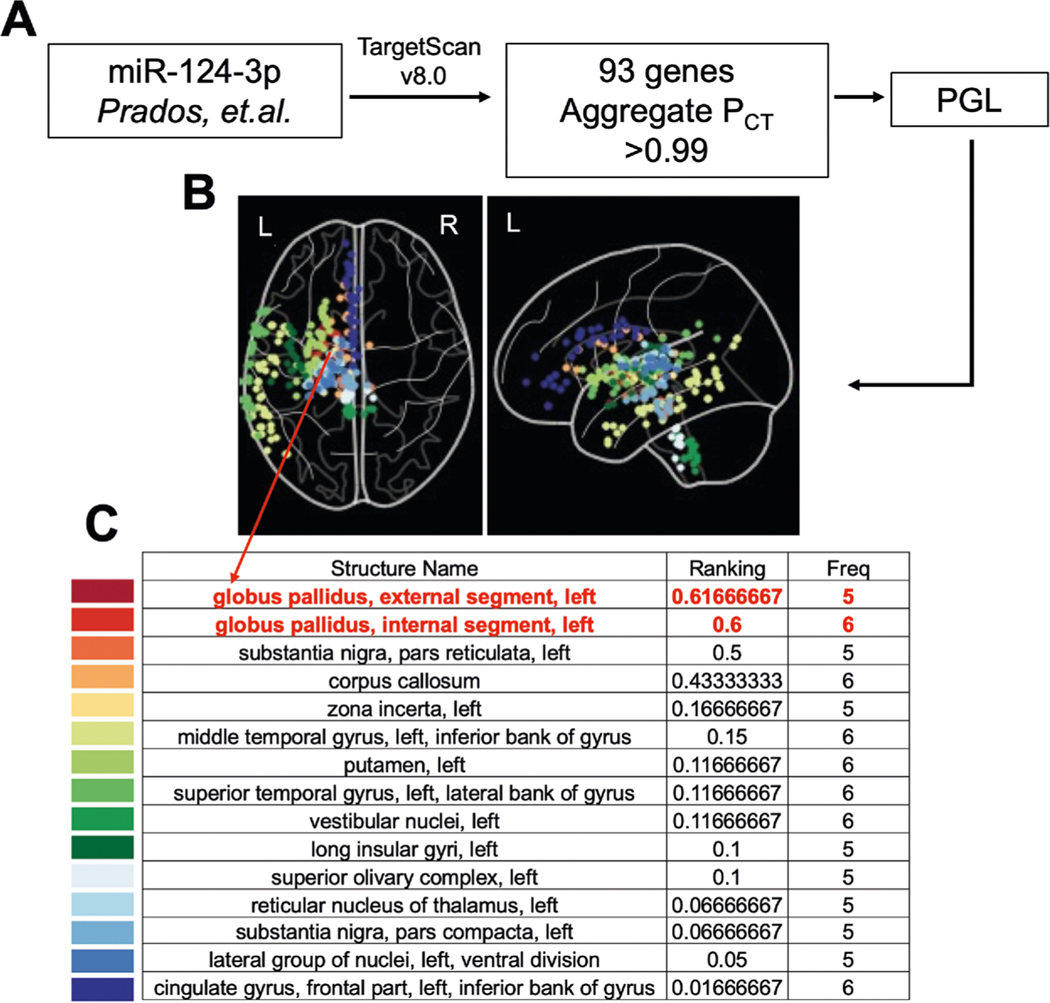
Targets of miR-124–3p used in process gene list (PGL) identify the globus pallidus (GP) as the top ROI. **A** Prados et al. described the miR-124–3p promoter region as hypomethylated in BPD. We isolated predicted messenger-RNA (mRNA) targets of miR-124–3p from TargetScan v 8.0 and refined the list based on the conserved binding sites in the 3′-utranslated regions (genes with an Aggregate Pct score of >0.99). **B, C** These 93 genes were utilized for PGL and a list of regions of interest were generated (regions with significant co-expression of the identified genes). We identified a list of regions, including the globus pallidus, external segment left, and internal segment left, as top regions of interest for further study. In each schematic of the brain representing the regions of interest identified, the color-coded points represent the regions as color-coded on the corresponding list below. Ranking: the importance of the ROI (see Supplementary Figs. 1, 2). Freq frequency, the number of times that the list of genes “points” to a certain region in the 6 brains available in the Allen Brain Atlas, only regions that appear as significant in five or six of the brains were used.

Hypomethylation of miR-124–3p was identified as associated with BPD and the severity of childhood maltreatment^10^. To investigate the role of miR-124–3p in our patient cohorts, we began by selecting miR-124–3p target genes with highly conserved preferential binding sequences (Aggregate P_CT_ scores >0.99; Supplementary Table 1). With this curated list of 93 total genes, 86 of which were in the Allen Atlas (Supplementary Tables 1 and 2, respectively), we applied PGL to determine the ROIs where miR-124–3p genes are highly co-expressed (Fig. 1A). From PGL, we obtained normalized expression of miR-124–3p targets in each brain region sampled by the Allen Human Brain Atlas^28^. We obtained a ranked (for a description, see Methods section 2.5) set of ROIs to be studied further (Fig. 1B, C). We identified the globus pallidus external segment left; globus pallidus, internal segment left; substantia nigra, pars reticulata left; corpus callosum, and others (Fig. 1B, C). To further validate the GP as the top ROI, we divided the list of miR-124–3p targets into two randomized sub-lists, which we then ran independently through PGL. The left GP (both internal and external) was identified as a top ROI from both lists of randomized miR-124–3p target genes (Supplementary Fig. 1 and Supplementary Table 2). Next, to assess the false positive rate of identification of ROIs from PGL, we generated 100 lists containing 30, 100, or 300 randomly selected genes. We observed that lists with 30 or 100 genes resulted in no false positive ROI, while the 100 lists of 300 genes resulted in six lists with false positives (Supplementary Fig. 2). Therefore, this finding not only suggests that GP co-expresses miR-124–3p target genes, but additionally provides assurance that our PGL approach is robust and reproducible.

### Differences in serum levels of miR-124–3p and volume of globus pallidus segment left are observed in BPD inpatients

**Fig. 2:**
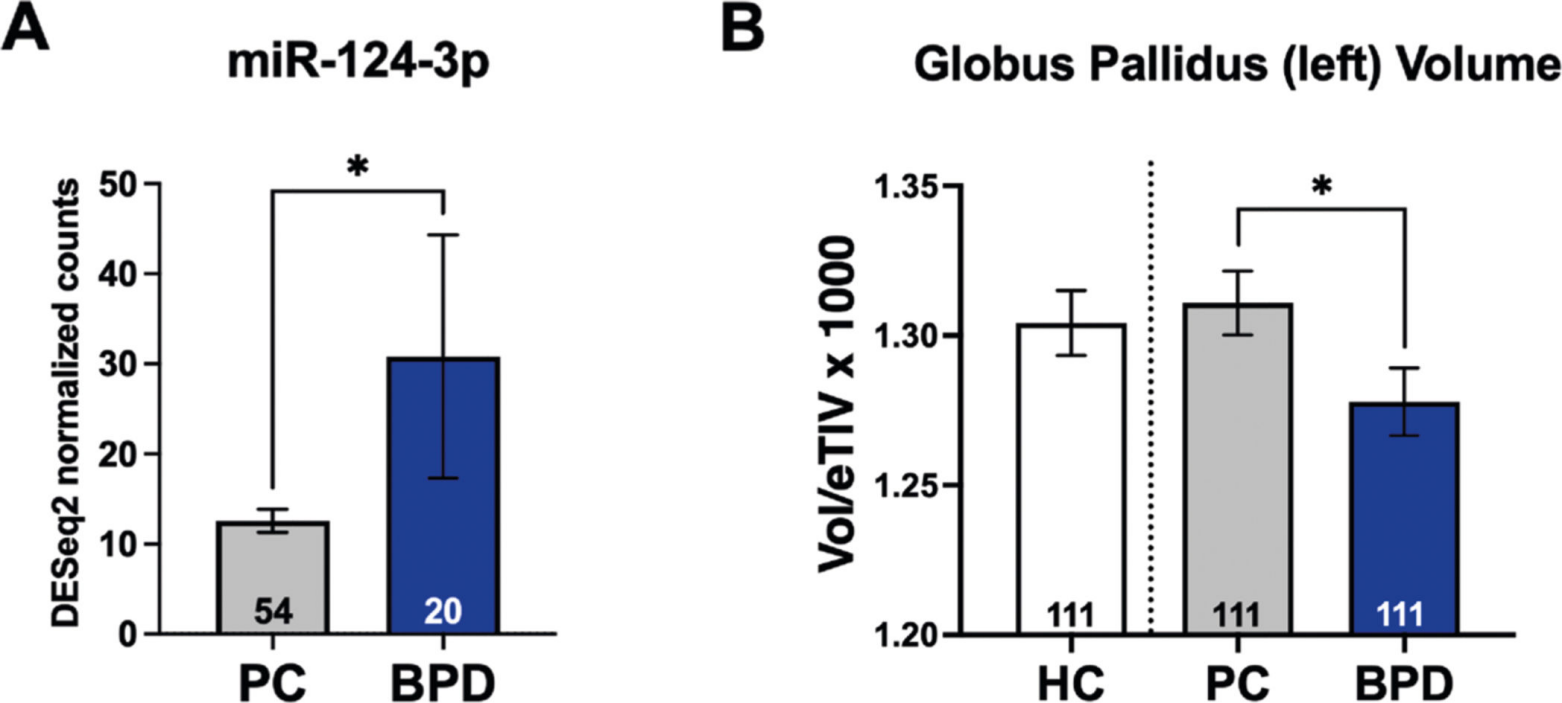
BPD patients have increased levels of circulating miR-124–3p and present and reduced left globus pallidus volume. Borderline personality disorder (BPD) and psychiatric control (PC) participants were in-patients at The Menninger Clinic, Houston, Texas. **A** Cell-free circulating miRNA levels were quantified, showing that miR-124–3p was significantly higher in the BPD group than in the PC (Wilcoxon rank-sum test, *p* = 0.034). **B** When comparing globus pallidus (GP) Left volume between BPD, PC, and healthy controls (HC), we found a significantly smaller volume in the left GP, which survived Bonferroni in the BPD group compared to PC (Wilcoxon rank-sum test, *p* < 0.0001). We were only interested in quantifying the BPD vs. PC comparison, the HC are shown only for visual comparison.

To initially detect differences in miR-124–3p levels in our BPD patient cohort, we isolated cell-free peripheral RNA and quantified miRNA expression levels from 54 PC and 24 BPD patients (Supplementary Methods). As expected with lower levels of methylation of miR-124–3p shown in BPD patients^10^, we observed that miR-124–3p levels were significantly higher in BPD than PC (Fig. 2A, Wilcoxon rank-sum test, *p* = 0.034). The removal of a possible outlier in the BPD group rendered a trend-level comparison (*p* = 0.065); however, given our sample size, it is likely that additional participants are needed. We found no significant difference in left GP volume between HC and PC (Fig. 2B, Unpaired *t*-test, *p* = 0.6584). Comparing the left GP volume between BPD and PC, we found a significantly smaller volume in the left GP in the BPD group (Fig. 2B, Wilcoxon rank-sum test *p* < 0.0001). Therefore, volumetric change of the left GP is a feature observable and specific to the BPD brain.

### Agreeableness but not neuroticism negatively correlates with globus pallidus volume

**Fig. 3:**
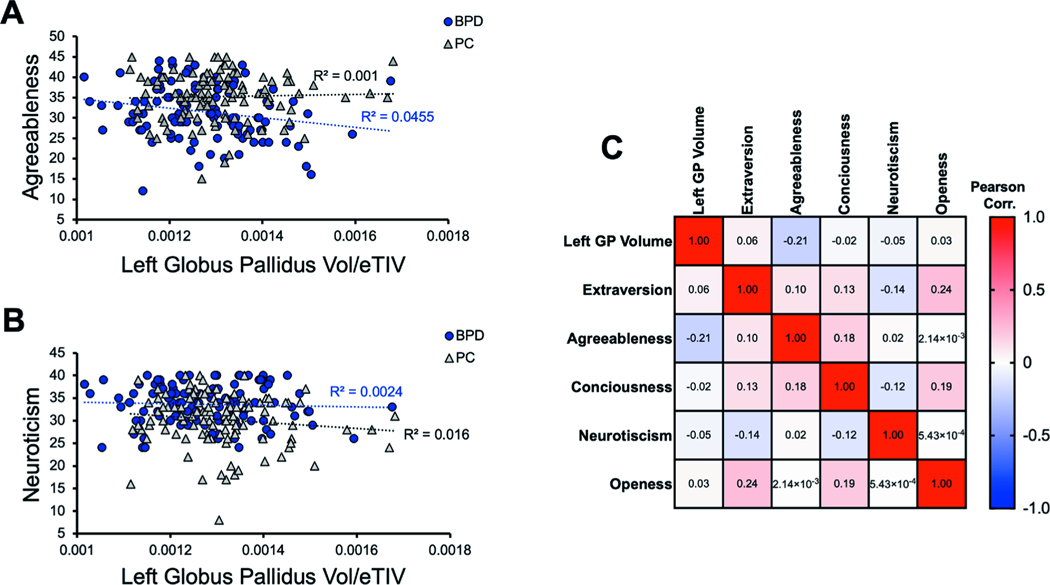
BFI personality traits of agreeableness negatively correlates with left globus pallidus (GP) volume in BPD, while neuroticism does not. **A** Correlation of left GP volume and agreeableness (*R*^2^ = 0.0455, linear regression *p* = 0.0245) in the BPD not in the PC group (*R*^2^ = 0.001, linear regression *p* = 0.746). **B** There was no significant correlation between left GP volume and neuroticism in the BPD cohort (*R*^2^ = 0.0024, linear regression *p* = 0.188) nor in the PC group (*R*^2^ = 0.016, linear regression *p* = 0.613). **C** Exploratory correlations of left GP volume and BFI traits of Extraversion, agreeableness, Consciousness, neuroticism, and Openness in BPD cohort.

As part of our patient cohort and the Menninger Outcomes project (30), we obtained admissions data from the BFI of Personality Dimensions in five categories of agreeableness, openness, extraversion, consciousness, and neuroticism. High neuroticism and low agreeableness are personality features observed in BPD^22^. Therefore, we considered these two correlations as our primary hypotheses and the other three BFI dimensions as exploratory. We observed that agreeableness was negatively correlated with left GP volume (*R*^2^ = 0.0455, linear regression *p* = 0.0245) while there was no correlation in the PC group (*R*^2^ = 0.001, *p* = 0.746; Fig. 3A). Interestingly, we observed that there was no significant correlation between left GP volume and neuroticism in our BPD cohort (*R*^2^ = 0.0024, *p* = 0.188) or in the PC group (*R*^2^ = 0.016, *p* = 0.613). With exploratory correlations utilizing Pearson’s multiple correlations matrix, we observed nonsignificant findings for openness, extraversion, or consciousness (Fig. 3C). Therefore, in our BPD patient cohort, left GP volume negatively correlated with agreeableness and not with neuroticism. Further studies are necessary to fully evaluate this novel association with left GP volume.

### Suicidal ideation recovery in BPD inpatients is negatively correlated with left Globus Pallidus volume

**Fig. 4:**
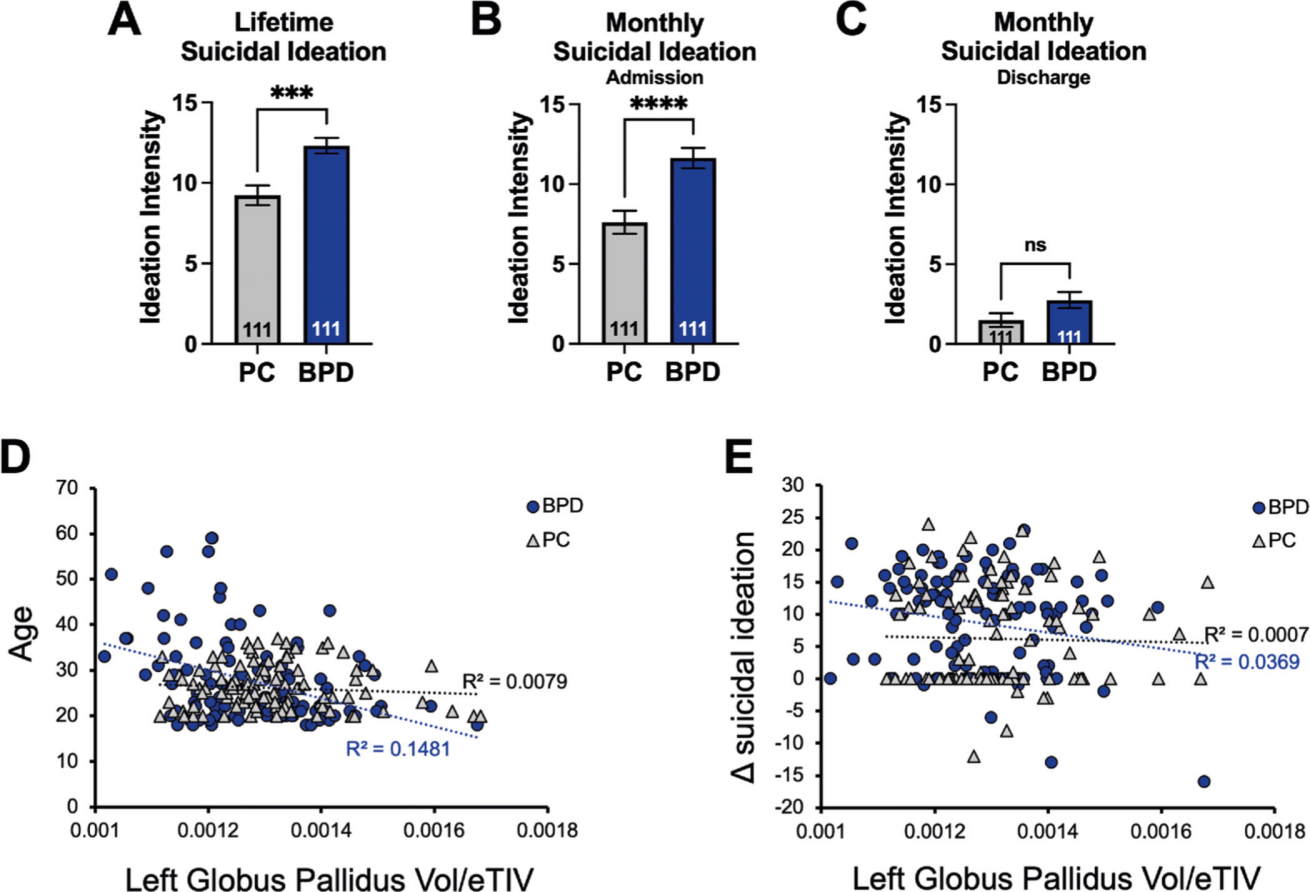
Suicidal ideation recovery in BPD inpatients is correlated with left globus pallidus (GP) volume. In our BPD cohort, **A** lifetime suicidal ideation intensity (Unpaired *t*-test, *p* = 0.0001) and **B** monthly suicidal ideation intensity at admission (Unpaired *t*-test, *p* < 0.0001) were significantly higher compared with the PC group. **C** At discharge, we observed a decrease in suicidal ideation intensity in both groups, and BPD was not statistically different from PC (Unpaired *t*-test, *p* = 0.0617). **D** There was a correlation between left GP volume and age in the BPD group (*R*^*2*^ = 0.1481, linear regression *p* = 5.34 × 10^−5^) that was not observed in the PC cohort (*R*^*2*^ = 0.0079, linear regression *p* = 0.3613). **E** Left GP volume was negatively correlated with recovery from suicidal ideation in BPD (*R*^*2*^ = 0.0369, linear regression *p* = 0.0443). No correlation between recovery from suicidal ideation and GP volume was observed in the PC cohort (*R*^*2*^ = 0.0007, linear regression *p* = 0.7929).

BPD patient populations have a high mortality rate, with 8–10% of patients dying by suicide^5^. In our BPD cohort, lifetime suicidal ideation (Fig. 4A, Unpaired *t*-test, *p* = 0.0001) and monthly suicidal ideation at admission (Fig. 4B, Unpaired *t*-test, *p* < 0.0001) were significantly higher compared to the PC group. At the time of discharge, we observed a decrease in suicidal ideation in our BPD group, which was not statistically different from the PC cohort (Fig. 4C, Unpaired *t*-test, *p* = 0.0617); however, a larger sample size may identify significant differences. To rule out any age-dependent effects in the left GP volume, we compared the distribution of age between BPD and PC cohorts. As expected by design, we did not observe a significant difference in age between the groups (not shown; Welch’s *t*-test, *p* = 0.1570). However, we observed a relationship between left GP volume and age in the BPD group

(Figure 4D; *R*^2^ = 0.1481, linear regression *p* = 5.34 × 10^−5^) that was not observed in the PC cohort (*R*^2^ = 0.0079, *p* = 0.3613). Although there was no statistical difference between mean age in the BPD and PC groups, the spread of the data was quite different in the BPD group. Removal of older participants to better match the distribution of PC (>40 years old, totaling 14 participants) from the BPD cohort did not extinguish the significant correlation between age and left GP volume (not shown; *R*^2^ = 0.1003, *p* = 0.0016). We asked if left GP volume was associated with recovery from suicidal ideation (Δ ideation intensity) in BPD and PC. We found that left GP volume was negatively correlated with recovery from suicidal ideation in BPD (*R*^2^ = 0.0369, *p* = 0.0443) but not in PC (*R*^2^ = 0.0007, *p* = 0.7929). Therefore, suicidal ideation recovery in BPD patients was associated with left GP volume, suggesting that left GP volume alteration is specific to BPD and related to suicide ideation recovery, as this is not observed in comorbidity-matched controls.

## DISCUSSION

BPD is characterized by pervasive patterns of affective instability, self-image disturbances, instability of interpersonal relationships, marked impulsivity, and suicidal ideation/attempt, causing significant distress and reducing the quality of life. Multiple studies have aimed to understand BPD etiology; however, these were limited in the ability to connect clinical, genetic/epigenetic, and brain imaging findings. Studies that include brain imaging tend not to include adequate controls (psychiatric controls; PC) to account for comorbidities; inclusion of a PC group is a major strength of our work^17^. The miRNA miR-124–3p was implicated in the path from early life adverse events to BPD in adulthood through methylation analysis^10^. Here, we identified brain regions that highly co-express genes regulated by miR-124–3p. We found that regions, where miR-124–3p target genes were highly co-expressed, were predominantly involved in sensorimotor processing, with the region of interest of highest ranking being the GP external left segment, followed by left GP internal (Fig. 1). Next, we found that the left GP of BPD was significantly smaller compared to PC. The BFI measure of agreeableness and the recovery from suicidal ideation after treatment negatively correlated with left GP volume in BPD but not PC. Therefore, smaller left GP correlated with improved outcomes in relevant BPD symptoms. This seemingly contradictory finding suggests that compensatory mechanisms could be involved. Our study is, to our knowledge, the largest brain imaging study conducted on BPD so far.

Epigenetic modifications in specific brain regions are associated with the sustained abnormalities of most mental disorders^29^. However, the influence of epigenetic mechanisms on personality disorders is not well understood. The studies currently available are limited not only in number, but also in the ability to connect regional brain changes, epigenetic changes, and clinical outcomes in BPD^30^. A genome-wide association (GWAS) study investigated BPD patients affected with high levels of childhood adversity or subjects with MDD and a history of childhood adversity. The methylation status of miR-124–3p was associated with both severities of childhood adversity (higher methylation) and with BPD (lower methylation)^10^. Our work follows and is in line with those findings as we observed higher circulating levels of miR-124–3p in BPD. We utilized Process Genes List (PGL)^11–13^ to link changes in epigenetic regulation via miRNAs with brain areas that may be important to psychiatric conditions. We identified the left GP external segment as a brain region where miR-124–3p targets are most highly co-expressed, followed by the left GP internal. We saw that left GP volume was significantly smaller in BPD than in PC patients. We speculate that a mechanism contributing to BPD etiology—either protective or pathological—occurs through increased levels of miR-124–3p and downregulation of target genes highly co-expressed in the GP. To our knowledge, our work is the first to link miRNA-modulated gene expression levels to identify brain regions significantly altered in psychiatry disorders.

Abnormal activity in different cortical regions and in basal ganglia are part of behavioral disorders^31^. For example, patients with attention-deficit/hyperactivity disorder (ADHD) have reduced metabolism in the prefrontal cortex and in the associative part of the striatum and in the pallidum^32–34^. However, little is known about the role of the basal ganglia, the GP, in personality disorders or BPD specifically. The GP is a central hub in the basal ganglia where the processing of motor and non-motor information occurs via connections with the other basal ganglia nuclei and brain regions^35,36^. Connections between the GP and brain regions involved in emotional processing have been described in ref. ^37^.

We showed that PGL is robust and reproducible at localizing regions of interest (Fig. 1 and Supplementary Figs. 1, 2) and it consistently identified the left GP as a likely target modifiable by miR-124–3p. Therefore, there might be a mechanism involving the basal ganglia and the limbic system, specifically in BPD. For example, a study showed that persons with BPD exhibited altered emotional perception from body movements in videos. BPD participants showed less confidence in their perception of depicted emotions, especially when these were difficult to identify^38^. Given the role of the GP in the control of movement, it is also possible that Neurological Soft Signs—neurologic anomalies only evidenced by specific motor, sensory, or integrative testing—that are altered in our BPD^39^, are influenced by GP volume. Future work on understanding the changes in functional connectivity in BPD should focus on regions involved in emotional processing and mechanisms that would be compromised from GP atrophy.

Using the BFI, Fowler et al. show that high neuroticism and low agreeableness scores were most associated with BPD^22,23^. Our work connected agreeableness, but not neuroticism, to changes in the left GP volume in BPD. Multiple factors could contribute to this finding. A less well-recognized role of the GP may be in emotional processing and behavior. Projections from the GP internal segment to the lateral habenula (LHb) have been described and may be critical for the reward system^37^, and we have shown that the habenula is a likely important brain locus in suicidality^12,16^. Future studies on BPD behaviors should consider focusing on the compensatory mechanisms converging on GP and LHb connectivity that may result in alleviated BFI personality features.

Patients with BPD stand out as having high rates of self-harm, suicide attempts (over 70% of patients), and death by suicide (8–12%)^40–42^. This significant risk for suicidal behaviors occurs independently of BPD’s common psychiatric comorbidities^43^. We identified the smaller left GP only in BPD, as compared to PC. This is of great significance given the connectivity of the GP to the LHb^37^ and the role of the LHb in suicidal ideation^44^. We found that left GP volume negatively correlated with recovery from suicidal ideation (Fig. 4E). It is tempting to speculate that a smaller GP may be associated with a protective compensatory mechanism − potentially acting through miR-124–3p through suppressed protein expression − in the GP and thereby contributing to recovery from suicidal ideation after treatment in BPD. Future work is needed to elucidate if this putative mechanism holds true.

BPD symptoms may become less prevalent with advancing age^45^. A Danish registry study found that for women with a BPD diagnosis the prospective diagnostic stability across a study period of 18 years was only 37%, confirming the potential for substantial variation in symptoms as patients age^46,47^. We found that smaller left GP volume negatively correlated with older age in the BPD cohort but not in PC (Fig. 4D). Furthermore, Grant and colleagues found a decrease in the prevalence of BPD across the lifespan, with the most notable change after 44 years of age^48^. We observed that by removing participants that were older than 40 years of age, the correlation between left GP and age remained (not shown). Therefore, changes in the left GP may serve as a protective compensatory mechanism specific to BPD that aids in recovery from suicidal ideation with treatment. Future studies should focus on identifying possible changes in functional connectivity between the left GP and the LHb, as well as other brain regions, in BPD and PC.

The principal limitation of neuroimaging studies is that causality is challenging to discern. We believe that studying brain regions genetically or epigenetically associated with a symptom or disorder helps alleviate this problem. Another common limitation in psychiatry research is that patients of any specific disorder share phenotypes and experiences (poor sleep, shame, and stigma) with patients of other disorders and these phenotypes may or may not be associated with brain parameters. In addition, given the low rates of psychotic features in patients that participated in the MIND-MB dataset, we were unable to include BPD patients with psychotic disorders. Future work in this area will be important to understanding BPD etiology further, given the putative striatal involvement in psychosis^49^. Matching BPD participants with a control group that presents with similar psychiatric comorbidities minimizes that possible problem and this is a major strength of our work. However, the high comorbidity among personality disorders, including in our sample, makes it challenging to study BPD in isolation. Another limitation is that the laterality observed in this study could be due to real differences between the right and left GP, or to the fact that the Allen Atlas sampled the left much more than the right side. Finally, although this study does not include genetics and is therefore appropriately powered as a brain imaging study, one of the main future directions is the study of the genes modulated by miR-124–3p and how they could modulate GP anatomy and function. For such study, we will likely need a larger sample.

In conclusion, using PGL with target genes regulated by miR-124–3p, we identified the left GP as a brain region where mRNA targets are significantly co-expressed. PGL is ideal for groups of genes working together, such as genes regulated by miRNAs. This is significant, as miRNAs are potentially druggable targets making PGL a possibly translatable approach from MRI findings. Accordingly, although more research is necessary before attempting translational studies, both miR-124–3p and the genes modulated by it are possible future targets to accelerate the improvement of BPD symptoms. Furthermore, we found that levels of serum miR-124–3p were significantly elevated in the BPD group, and the left GP was significantly smaller compared to PC. Although we unexpectedly observed a negative correlation between agreeableness and left GP volume, it is tempting to speculate this change in agreeableness is part of the recovery process from suicidal ideation, as small left GP volume negatively correlated with recovery from suicidal ideation. Therefore, our study is the first to implement PGL—a high-throughput, robust, and reproducible approach to identify brain regions—to BPD and from which we could identify differences in brain regions separating BPD from other related psychiatric conditions.

## Supplementary Material

Aloi supple

## Figures and Tables

**Table 1.** Demographics of borderline personality disorder (BPD), psychiatric control (PC), and healthy control (HC) participants.

Characteristics	Borderline personality disorder (BPD, *N* = 111)	Psychiatric controls (PC, *N* = 111)	Healthy controls (HC, *N* = 111)	*p* value (BPD vs. PC vs. HC)

Demographics				
Mean age ± standard dev.	27.61 ± 9.67	26.16 ± 4.71	26.50 ± 5.75	
Female	46%	54%	54%	0.3162
Male	54%	46%	46%	0.4257
Major comorbidities	Borderline personality disorder (BPD, *N* = 111)	Psychiatric controls (PC, *N* = 111)		*p* value (BPD vs. PC)
Depression^#^	66%	54%		0.0547
Anxiety^$^	29%	22%		0.2164
PTSD^%^	19%	20%		0.8651
Bipolar (I, II, and other)^&^	24%	26%		0.8208
Personality disorders	Borderline personality disorder (BPD, *N* = 111)	Psychiatric controls (PC, *N* = 111)		*p* value (BPD vs. PC)
Avoidant	30%	18%		0.0406
Obsessive-compulsive	22%	7%		0.0022
Narcissistic	5%	3%		0.3073
Anti-social	7%	2%		0.0522
Schizoid*	0%	0%		N/A
Borderline*	100%	0%		N/A

Participants were inpatients at The Menninger Clinic throughout the remainder of the study. There were no significant differences in age (one-way ANOVA) or sex (Chi-square test) between the three groups. There were no significant differences among major comorbidities between BPD and PC groups (Chi-square test

# includes multiple measures of depression

$ includes generalized anxiety

% includes past and present PTSD

& includes Bipolar I, II, and others). Since personality disorders are highly comorbid, we quantified SCID-II measures of personality disorders and compared the BPD and PC groups. There were significant differences in comorbid personality disorders between BPD and PC groups in avoidant and obsessive-compulsive personality disorders (Chi-square test

* excluded from Chi-square test).

## Data Availability

The data that support the findings of this study are available from the corresponding author R.S. upon reasonable request.
