# Supplementary material for: miR-124-3p target genes identify globus pallidus role in suicide ideation recovery in borderline personality disorder
Source: Npj Ment Health Res. 2023 Jun 5;2:8. doi: 10.1038/s44184-023-00027-w (PMC10500603; doi:10.1038/s44184-023-00027-w)
Supplement: Supplementary file 2 — Supplementary Data [file 44184_2023_27_MOESM2_ESM.pdf]

Supplementary Figure 1

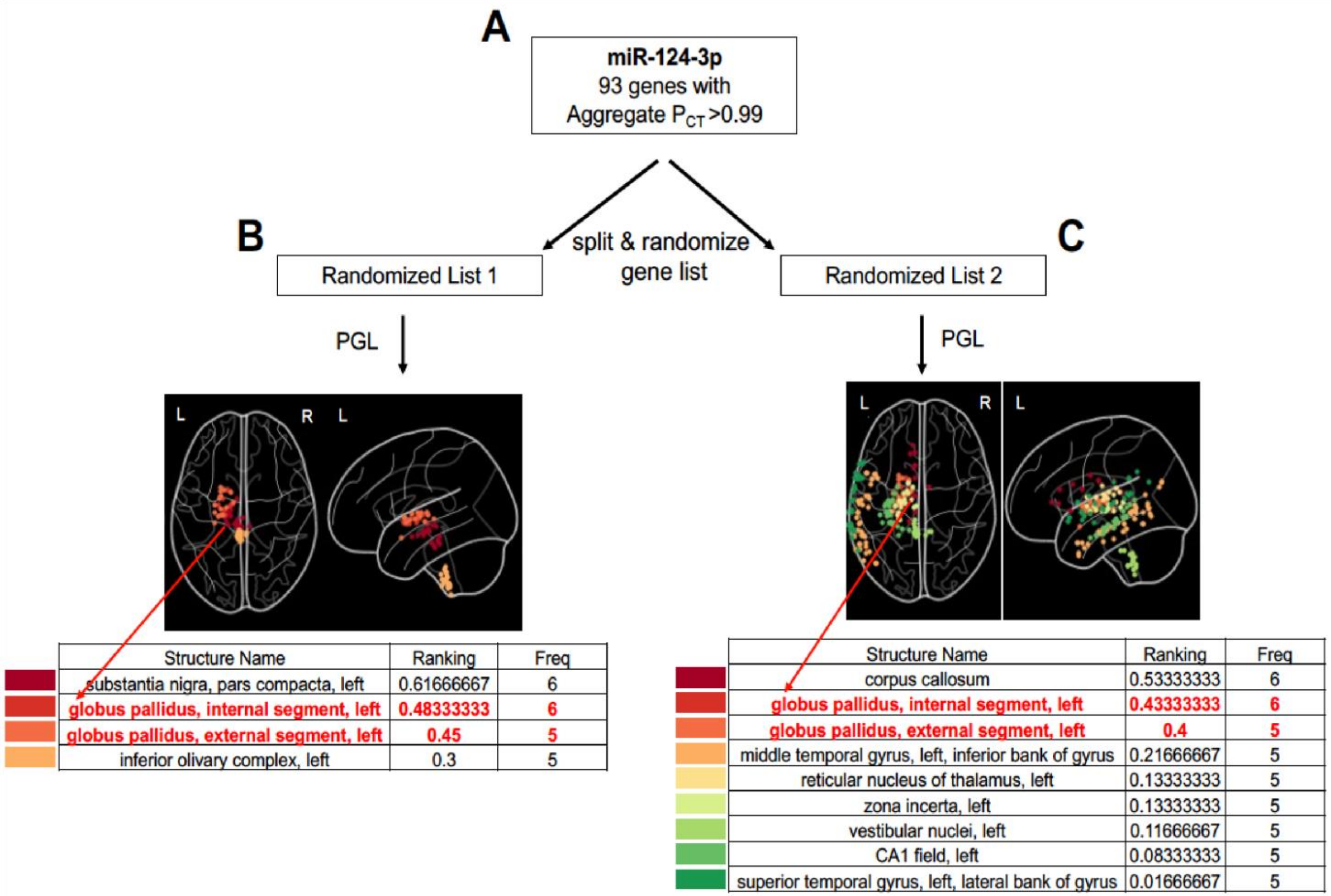

Structure Name	Ranking	Freq
substantia nigra, pars compacta, left	0.61666667	6
globus pallidus, internal segment, left	0.48333333	6
globus pallidus, external segment, left	0.45	5
inferior olivary complex, left	0.3	5

Structure Name	Ranking	Freq
corpus callosum	0.53333333	6
globus pallidus, internal segment, left	0.43333333	6
globus pallidus, external segment, left	0.4	5
middle temporal gyrus, left, inferior bank of gyrus	0.21666667	5
reticular nucleus of thalamus, left	0.13333333	5
zona incerta, left	0.13333333	5
vestibular nuclei, left	0.11666667	5
CA1 field, left	0.08333333	5
superior temporal gyrus, left, lateral bank of gyrus	0.01666667	5

Supplementary Figure 1: Randomization of miR-124-3p targets to test reproducibility and robustness of Process-Gene List (PGL) identify the Globus Pallidus as the top ROI in both sub-lists of genes. A) We collected predicted messenger-RNA (mRNA) targets of miR-124-3p from TargetScan (v 8.0) and refined the list based on the conserved binding sites in the 3'-utranslated regions (or Aggregate Pct score of >0.99). We then randomized and split the list into two, generating **B)** Randomized list 1 and **C)** Randomized list 2 to be used for PGL analysis. These two lists of genes were utilized in PGL and a list of ROIs was generated for each. We identified a list of regions including the globus pallidus, external segment left, and internal segment left, as top regions of interest. Importantly, only the pallidum was identified in both sub-lists, showing the strongest significance. In each schematic of the brain representing the regions of interest identified, the color-coded points represent the regions as color-coded on the corresponding list below.

Supplementary Figure 2

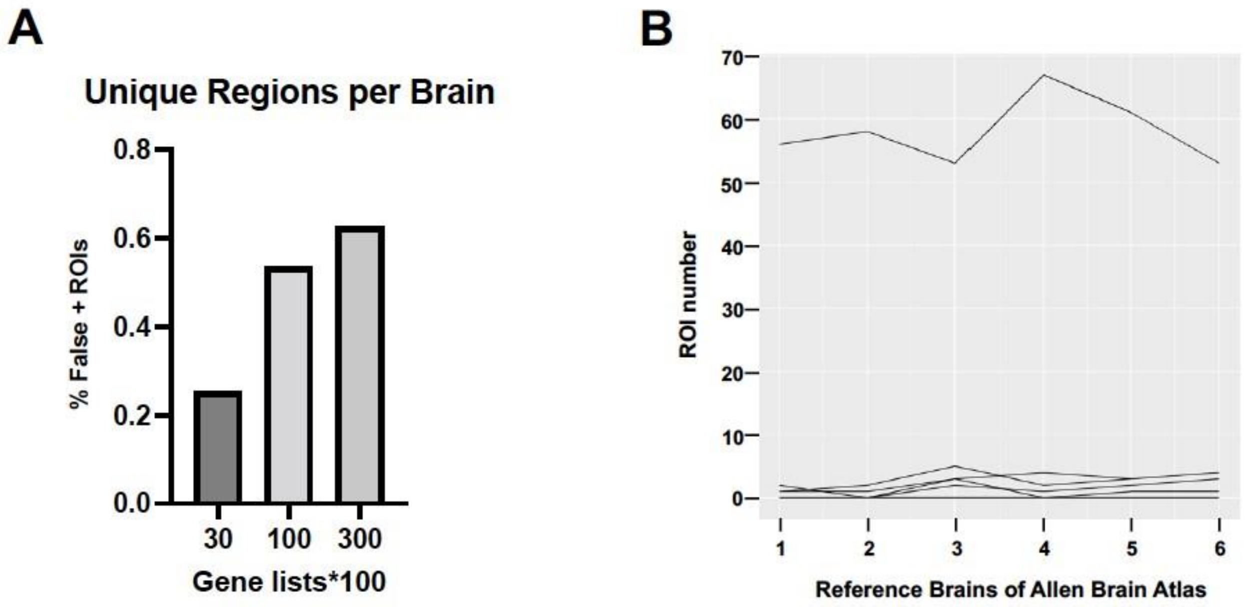

Supplementary Figure 2: PGL is robust and reproducible, yielding minimal false-positive brain regions of interest (ROIs). To study the possibility that PGL could yield false positive ROIs, we created 100 lists with 30 random genes, 100 lists with 100 random genes, and 100 lists 300 random genes. We then applied PGL with permissive conditions (unique ROIs that appeared in a single reference brain). **A)** Utilizing PGL with 100 lists of 30 randomly chosen genes, we found 0.25% false positive ROIs (355 of 140,500 comparisons). PGL with 100 lists of 100 genes yielded 0.54% false positive ROIs (752 of 140,500 comparisons), while PGL with 100 lists of 300 genes yielded 0.62% (880 of 140,500 comparisons) false positive ROIs. False-positive ROIs were identified by taking only Wilcoxon/Bonferroni into account on individual brains. However, with all 100 lists of 30 or 100 randomly chosen genes, no ROIs were identified when the criteria of identifying an ROI in 5 or 6 of the individual brains (as we did with the miR-124-3p list of genes) was also added. **B)** From the 100 lists of 300 randomly chosen genes, only 6 lists yielded false positive ROIs; 5 lists yielded one or two regions only (with large *p* values compared to miR-124-3p target gene lists), and one had many regions, with low *p* values. We conclude that using our two chosen conditions – Wilcoxon and Bonferroni, plus ROIs identified in at least 5 of 6 reference brains available in the Allen Brain Atlas – only larger lists of genes would eventually yield a false positive that could be misconstrued for a true result. Lists of ~100 genes like the one used in miR-124-3p are expected to yield no false positives.

Supplementary Table 1: Gene targets of miR-124-3p used in Process-Gene List (PGL) identify the Globus Pallidus (GP) as top ROI. The 93 target genes of miR-124-3p with Aggregate P_{CT} score >0.99 were downloaded from Human TargetScan v 8.0. This list was used for PGL to identify the GP as a top ROI.

Target gene	Gene name	Number of 3P-seq tags supporting UTR + 5
PLEKHM3	pleckstrin homology domain containing, family M, member 3	11
ZBTB20	zinc finger and BTB domain containing 20	5
NFATC1	nuclear factor of activated T-cells, cytoplasmic, calcineurin-dependent 1	74
CTDSP1	CTD (carboxy-terminal domain, RNA polymerase II, polypeptide A) small phosphatase 1	1299
QSER1	glutamine and serine rich 1	34
MAGT1	magnesium transporter 1	937
CHIC1	cysteine-rich hydrophobic domain 1	16
TTL	tubulin tyrosine ligase	442
PIK3C2A	phosphatidylinositol-4-phosphate 3-kinase, catalytic subunit type 2 alpha	280
LRRC1	leucine rich repeat containing 1	343
LRRC58	leucine rich repeat containing 58	940
KIAA0247	KIAA0247	161
ATL3	atlastin GTPase 3	734
WIPF2	WAS/WASL interacting protein family, member 2	214
VAMP3	vesicle-associated membrane protein 3	515
FLOT2	flotillin 2	37
QKI	QKI, KH domain containing, RNA binding	323
TRAF3	TNF receptor-associated factor 3	833
NFIC	nuclear factor I/C (CCAAT-binding transcription factor)	26
FRMD4B	FERM domain containing 4B	12
GGA2	golgi-associated, gamma adaptin ear containing, ARF binding protein 2	645
TEAD1	TEA domain family member 1 (SV40 transcriptional enhancer factor)	740
LAMC1	laminin, gamma 1 (formerly LAMB2)	1082
G3BP1	GTPase activating protein (SH3 domain) binding protein 1	688
PLXNA3	plexin A3	2992
TUB	tubby bipartite transcription factor	743
OSBP	oxysterol binding protein	101
STRN	striatin, calmodulin binding protein	476
B4GALT1	UDP-Gal:betaGlcNAc beta 1,4- galactosyltransferase, polypeptide 1	2265
CD164	CD164 molecule, sialomucin	2038
RNPEPL1	arginyl aminopeptidase (aminopeptidase B)-like 1	239
MYO10	myosin X	3866
C1GALT1	glycoprotein-N-acetylgalactosamine 3-beta-galactosyltransferase, 1	44
CPT1A	carnitine palmitoyltransferase 1A (liver)	869
MITF	microphthalmia-associated transcription factor	593
AL626787.1		5
OSBPL3	oxysterol binding protein-like 3	200
EYA4	eyes absent homolog 4 (Drosophila)	242
SERP1	stress-associated endoplasmic reticulum protein 1	3242
ATP7A	ATPase, Cu++ transporting, alpha polypeptide	141
NFIX	nuclear factor I/X (CCAAT-binding transcription factor)	24
B4GALNT3	beta-1,4-N-acetyl-galactosaminyl transferase 3	5
CBX2	chromobox homolog 2	1816
GALNT10	UDP-N-acetyl-alpha-D-galactosamine:polypeptide N-acetylgalactosaminyltransferase 10 (GalNAc-T10)	3445
CC2D1B	coiled-coil and C2 domain containing 1B	42
SNTB2	syntrophin, beta 2 (dystrophin-associated protein A1, 59kDa, basic component 2)	254
GINM1	glycoprotein integral membrane 1	428
CBL	Cbl proto-oncogene, E3 ubiquitin protein ligase	309

TMEM104	transmembrane protein 104	657
ENAH	enabled homolog (Drosophila)	415
GXYLT1	glucoside xylosyltransferase 1	126
PHF19	PHD finger protein 19	490
MIB1	mindbomb E3 ubiquitin protein ligase 1	897
EVI5	ecotropic viral integration site 5	172
YY1	YY1 transcription factor	1803
VPS37C	vacuolar protein sorting 37 homolog C (S. cerevisiae)	3267
KLHL24	kelch-like family member 24	143
RASSF5	Ras association (RalGDS/AF-6) domain family member 5	27
OSBPL8	oxysterol binding protein-like 8	141
SRGAP1	SLIT-ROBO Rho GTPase activating protein 1	64
PTBP2	polypyrimidine tract binding protein 2	70
KIAA1671	KIAA1671	549
KLF13	Kruppel-like factor 13	5
PTPN12	protein tyrosine phosphatase, non-receptor type 12	167
CREBRF	CREB3 regulatory factor	24
RFX3	regulatory factor X, 3 (influences HLA class II expression)	37
RALGPS2	Ral GEF with PH domain and SH3 binding motif 2	201
CTDSPL	CTD (carboxy-terminal domain, RNA polymerase II, polypeptide A) small phosphatase-like	372
SEMA6A	sema domain, transmembrane domain (TM), and cytoplasmic domain, (semaphorin) 6A	106
TET3	tet methylcytosine dioxygenase 3	1437
NAT8L	N-acetyltransferase 8-like (GCN5-related, putative)	220
CPD	carboxypeptidase D	1766
XPO4	exportin 4	718
NEGR1	neuronal growth regulator 1	28
ONECUT3	one cut homeobox 3	5
RHOQ	ras homolog family member Q	179
PRKD1	protein kinase D1	75
CTNND1	catenin (cadherin-associated protein), delta 1	743
SNX18	sorting nexin 18	174
SH3PXD2A	SH3 and PX domains 2A	152
ZFP36L2	ZFP36 ring finger protein-like 2	317
SREK1	splicing regulatory glutamine/lysine-rich protein 1	316
ELK3	ELK3, ETS-domain protein (SRF accessory protein 2)	2719
SLC39A9	solute carrier family 39, member 9	824
MYRIP	myosin VIIA and Rab interacting protein	9
SNAI2	snail family zinc finger 2	1233
UNC119B	unc-119 homolog B (C. elegans)	5
VAT1	vesicle amine transport 1	2844
EML6	echinoderm microtubule associated protein like 6	39
RAP2B	RAP2B, member of RAS oncogene family	594
CEBPA	CCAAT/enhancer binding protein (C/EBP), alpha	12449
FAR1	fatty acyl CoA reductase 1	814

Supplementary Table 2: Randomized target genes of miR-124-3p utilized to validate the Globus Pallidus (GP) as top ROI identified through ProcessGenesList (PGL). Of the 93 miR-124-3p target genes retrieved from Human TargetScan v 8.0, 86 were matched to genes reported by the Allen Brain Atlas. The 86 genes were randomized and divided to generate Random Gene List 1 (left) and Random Gene List 2 (right). See Supplementary Figure 1.

Target Gene List 1	Target Gene List 2
CBL	LAMC1
CPD	MYO10
CPT1A	OSBP
CEBPA	PIK3C2A
ELK3	PRKD1
B4GALT1	RFX3
FLOT2	TRAF3
NFIX	YY1
RAP2B	VAMP3
SNAI2	QKI
STRN	KIAA0247
TEAD1	GGA2
G3BP1	MYRIP
VAT1	KLF13
RHOQ	SLC39A9
ATL3	PLXNA3
ZBTB20	C1GALT1
SERP1	RNPEPL1
KLHL24	CTDSP1
TMEM104	SEMA6A
GALNT10	MAGT1
PTBP2	FAR1
XPO4	OSBPL8
SRGAP1	WIPF2
MIB1	TTL
CBX2	B4GALNT3
SNX18	NEGR1
NAT8L	PHF19
MITF	EYA4
OSBPL3	EVI5
RALGPS2	RASSF5
ENAH	CTDSPL
NFIC	SH3PXD2A
TUB	NFATC1
EML6	CC2D1B
CHIC1	LRRC1
VPS37C	LRRC58
SNTB2	QSER1
TET3	CD164
FRMD4B	KIAA1671
CTNND1	ONECUT3
ATP7A	UNC119B
	PTPN12
	ZFP36L2

Supplementary Methods

RNA isolation, library preparation, and miRNA sequencing

We collected 10 ml of whole blood (EDTA anticoagulant) and processed it within one hour using the Gentra Puregene blood kit (Qiagen, Germantown, MD) per the manufacturer's protocol. Blood was spun at 2,500xg for 10 minutes. Plasma was then removed and stored at -80° C until analysis. We extracted total RNA from plasma using the Qiagen miRNeasy serum/plasma kit (Qiagen, Germantown, MD). Concentration of total collected miRNA was quantified using an Agilent 2100 bioanalyzer (Santa Clara, CA). Sequencing libraries were constructed using the Qiagen Qiaseq miRNA library kit (Qiagen) according to the manufacturer's protocol. Library quality was assessed using the Agilent 4200 tape station to ensure the miRNA-sized library was approximately 180 nt in length. The sample was loaded onto an Illumina Nextseq500 instrument (San Diego, CA) and the miRNA-sized library was sequenced.

Sequencing data analysis

We used the global miRNA expression profiles to create a list of expressed miRNAs. The raw sequencing reads were trimmed using Cutadapt (Lindgreen, 2012) to remove adapter, primer, and poly-A sequences. Reads with fewer than 15 nt were discarded. Identification of known miRNAs were confirmed using miRbase v21 (Kozomara, et. al., 2014) and miRdeep2 v2.0.0.8 (Friedländer *et al.*, 2008, 2012). A miRNA expression matrix was constructed using the number of reads mapped to each miRNA. We determined differentially expressed (DE) miRNAs using the R package DESeq2 (v1.26.0) (Love, et.al., 2014). We considered miRNAs with DESeq2 normalized counts >10, which occurred in at least 50% of libraries to be expressed and we used these miRNAs in DE and for downstream analyses. We considered differences in miRNA expression between admission and SI recovery significant at Benjamini and Hochberg corrected $p < 0.05$ and $|\log_2 \text{Fold Change}| > 0.25$.
